# Comparison between dedicated MRI and symphyseal fluoroscopic guided contrast agent injection in the diagnosis of cleft sign in athletic groin pain and association with pelvic ring instability

**DOI:** 10.1007/s00330-023-09666-1

**Published:** 2023-05-05

**Authors:** Norman Holl, Judith Sarah Gerhardt, Thomas Tischer, Jens Krüger, Andres Arevalo-Hernandez, Robert Lenz, Marc-André Weber

**Affiliations:** 1 Institute of Diagnostic and Interventional Radiology, Paediatric and Neuroradiology, University Medicine Rostock, Ernst-Heydemann-Str. 6, 18057 Rostock, Germany; 2 Department of Orthopaedics, Rostock University Medical Center, Doberanerstr. 142, 18057 Rostock, Germany; 3 Sportchirurgische Praxis Dr. Jens Krüger, Potsdamer Straße 132, 10783 Berlin, Germany

**Keywords:** MRI, Symphysography, Groin pain, Athletes, Cleft injuries

## Abstract

**Objective:**

To compare dedicated MRI with targeted fluoroscopic guided symphyseal contrast agent injection regarding the assessment of symphyseal cleft signs in men with athletic groin pain and assessment of radiographic pelvic ring instability.

**Methods:**

Sixty-six athletic men were prospectively included after an initial clinical examination by an experienced surgeon using a standardized procedure. Diagnostic fluoroscopic symphyseal injection of a contrast agent was performed. Additionally, standing single-leg stance radiography and dedicated 3-Tesla MRI protocol were employed. The presence of cleft injuries (superior, secondary, combined, atypical) and osteitis pubis was recorded.

**Results:**

Symphyseal bone marrow edema (BME) was present in 50 patients, bilaterally in 41 patients and in 28 with an asymmetrical distribution. Comparison of MRI and symphysography was as followed: no clefts: 14 cases (MRI) vs. 24 cases (symphysography), isolated superior cleft sign: 13 vs. 10, isolated secondary cleft sign: 15 vs. 21 cases and combined injuries: 18 vs. 11 cases. In 7 cases a combined cleft sign was observed in MRI but only an isolated secondary cleft sign was visible in symphysography. Anterior pelvic ring instability was observed in 25 patients and was linked to a cleft sign in 23 cases (7 superior cleft sign, 8 secondary cleft signs, 6 combined clefts, 2 atypical cleft injuries). Additional BME could be diagnosed in 18 of those 23.

**Conclusion:**

Dedicated 3-Tesla MRI outmatches symphysography for purely diagnostic purposes of cleft injuries. Microtearing at the prepubic aponeurotic complex and the presence of BME is a prerequisite for the development of anterior pelvic ring instability.

**Clinical relevance statement:**

For diagnostic of symphyseal cleft injuries dedicated 3-T MRI protocols outmatch fluoroscopic symphysography. Prior specific clinical examination is highly beneficial and additional flamingo view x-rays are recommended for assessment of pelvic ring instability in these patients.

**Key Points:**

*• Assessment of symphyseal cleft injuries is more accurate by use of dedicated MRI as compared to fluoroscopic symphysography.*

*• Additional fluoroscopy may be important for therapeutic injections.*

*• The presence of cleft injury might be a prerequisite for the development of pelvic ring instability.*

## Introduction

Athletic groin pain represents a wide spectrum of possible underlying pathologies that often occur in response to chronic repetitive stress applied to healthy bone. Due to the anatomical complexity of the groin area, a variety of causes of groin pain might influence and delay the exact diagnosis, possibly leading to delayed targeted therapy. Among those conditions causing groin pain in athletes the incidence of osteitis pubis (OP), a noninfectious inflammation of the pubic bone, has been reported as high as 10–18% of injuries per year in soccer players [1], possibly causing a prolonged absence from sports. Accompanying injury patterns such as secondary and superior cleft might occur and are well recognized in the pathogenesis of groin pain [2, 3]. Furthermore, the presence of symphyseal cleft injuries was found to be associated with a delayed time to return to play [4]. Mechanistically, an increased sporty load exerts considerable stress on the pubic symphysis [5]. Especially the inherent high mechanical demands of multidirectional sports (e.g. soccer) on the pubic symphysis and its supporting musculoskeletal structures may increase the probability of overuse injuries such as OP.

The diagnostic approach to OP and associated pathologies involve clinical examination, clinical history, sports anamnesis, and imaging techniques. The latter favors the use of magnetic resonance imaging (MRI), which has been shown to reliably depict the pattern of injuries around the pubic symphysis involving the rectus abdominis and the adductor tendon origin [6]. Especially in younger patients, MRI is generally preferred over computed tomography (CT) due to the lack of ionizing radiation and superior imaging of surrounding soft tissue and possible bone inflammation. However, contrast agent injection guided by radiography, fluoroscopy, or CT imaging might be alternative approaches for the diagnosis of symphyseal cleft injuries. In that regard, symphyseal cleft injections might add to the diagnostic yield gained from MRI by possibly identifying the source the pain derives from [7]. In the case of OP, there is an accumulating body of evidence that after injection of corticosteroids and local anesthetics, the clinical symptoms improve, which might add important additional information to the diagnostic process [8]. However, given the fact that groin pain is a multifaceted pathology, the diagnostic approach has to consider multiple possibly causing pathologies. Brennan et al [9] compared MRI and radiography with additional contrast agent injection in the diagnosis of secondary cleft sign and found 100% sensitivity and specificity for both modalities. McArthur et al [10] retrospectively compared symphyseal CT arthrography and MRI and reported the CT arthrography to be advantageous for the detection of secondary cleft and tendon tears at the adductor origin as compared to MRI. Well in line with these findings are reports by Hopp et al [11], who found symphysography to be superior to MRI in the detection of symphyseal cleft injuries. Additionally, Murphy et al [3] employed symphysography as the gold standard for the detection of symphyseal cleft injuries. Other studies highlight the role of MRI not only in the diagnostics of groin pain [1] but also in evaluating the prognosis [12].

Therefore, the purpose of this study was to compare a dedicated 3-T MRI protocol with targeted fluoroscopy-guided symphyseal contrast agent injection regarding the assessment of symphyseal cleft injuries in men with athletic groin pain. We further assessed pelvic ring instability by use of radiography (“flamingo-view”).

## Methods

### Patient selection and demographic characteristics

66 male sport-active patients were prospectively examined. For each patient, all examinations were done within one session on the same day. All athletes were referred to our clinic by a highly specialized groin surgeon in private practice after an initial clinical examination using a standardized procedure. All referred patients presented with characteristic groin pain and after clinical examination were suspected of secondary or superior cleft injuries. The level of activity differed between patients ranging from professional to recreational athletes (Table 1). Inclusion criteria were (1) male athletes with a history of groin pain, (2) suspected cleft injury after standardized clinical examination, (3) no prior surgical treatment of cleft- or adductor injuries.

**Table 1:** Demographics and patient characteristics

Number of patients	66
Mean age [years]	31.7 *± *10.5
Mean height [cm]	183 ± 4.3
Mean weight [kg]	80 ± 7.2
Type of sport activity	
Soccer	56
Other (including ice hockey, running, cycling)	10
Level of sport activity	
Professional	13
Semi-professional	8
Recreational	45
Average training sessions per week	4.2 ± 1.6
Mean duration of symptoms [months]	12 ± 12.5 months
Side of Symptoms	
Right	28
Left	26
Both sides	11
Central	1
Previous therapy? (including NSAIDs, Physical therapy, Local injections)	64
Trigger Event known	
Yes	14
No	52

Our local Ethical Committee approved the present protocol and informed consent was obtained from all patients. The study was approved by the Ethical Committee of Rostock University (approval No. A 2020-0040).

### Magnetic resonance imaging: acquisition and analysis

**Fig. 1:**
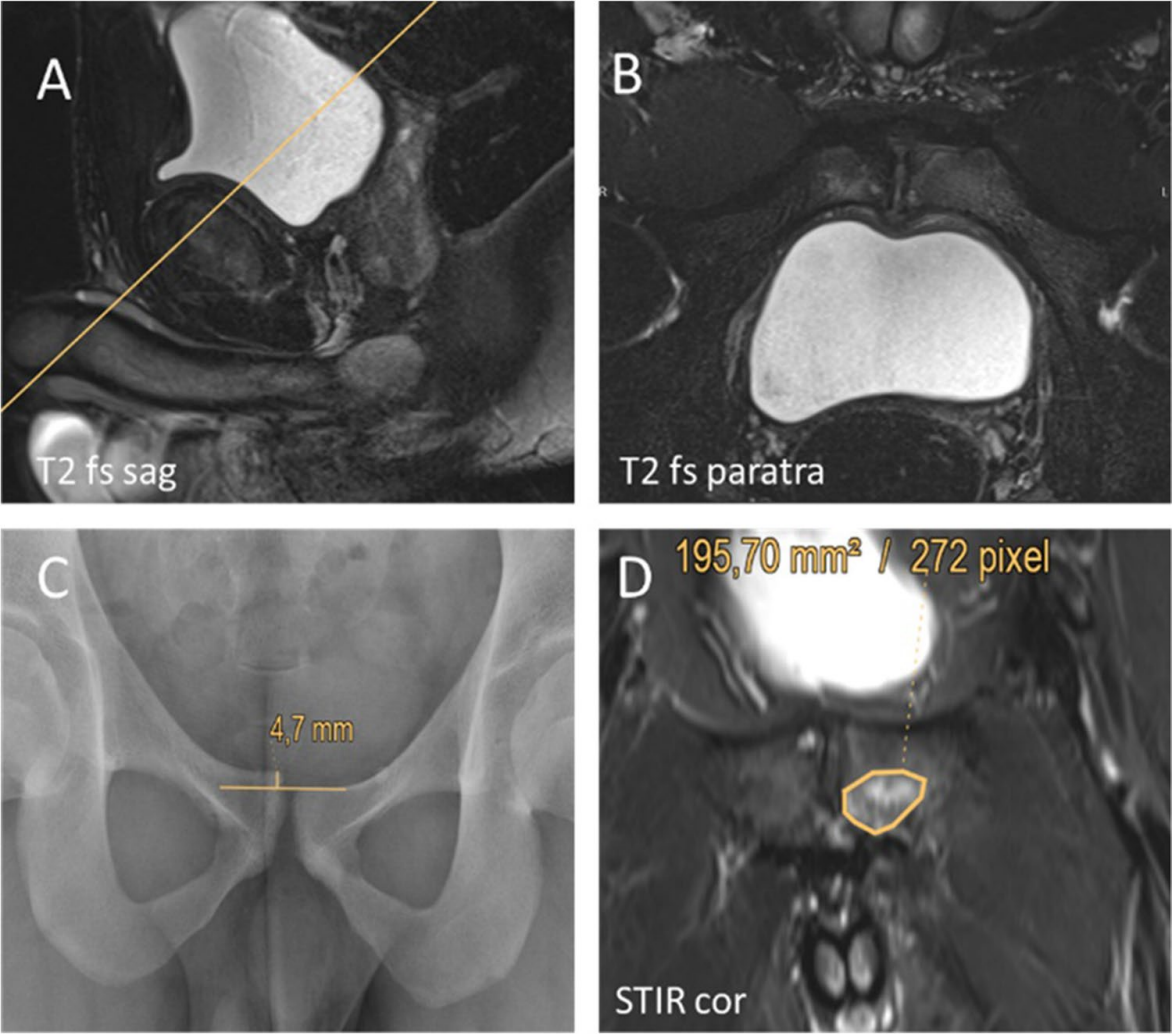
Example of orientation angle for paratransversal sequences (yellow line in **A**) and corresponding paratransversal image (**B**). Measurement of anterior pelvic ring instability (**C**) and maximal area of bone marrow edema (**D**)

MRI was performed using a 3-Tesla whole-body system (Magnetom Skyra Fit, Siemens Healthineers) and an 18-channel body matrix coil strapped over the pelvic area. Table 2 lists the specifics of the acquired sequences of the dedicated symphyseal MRI protocol. The orientation angle for paratransversal sequences is shown in Fig. 1.

**Table 2:** 3-T dedicated symphyseal MRI protocol and sequence specifics

*Sequence*	*Orientation*	TR (ms)	TE (ms)	*Slice thickness (mm)*	*Field of view (mm)*
T2 tirm	*Coronal*	4000	72	3	380
T1 tse	*Coronal*	600	10	3	380
T2 tse fs	*Sagittal*	5000	106	2	240
T2 tse fs	*Transversal*	5690	108	3	200
T2 tse fs	*Paratransversal*	7460	109	2	200
T1 tse	*Paratransversal*	582	11	2	200
T2 tse	*Paratransversal*	7070	109	2	200

Abbreviations: *fs* = fat saturation, *tse* = turbo spin echo, *tirm* = turbo inversion recovery magnitude

### Diagnostic criteria of MRI, X-ray, and fluoroscopic findings

**Fig. 2:**
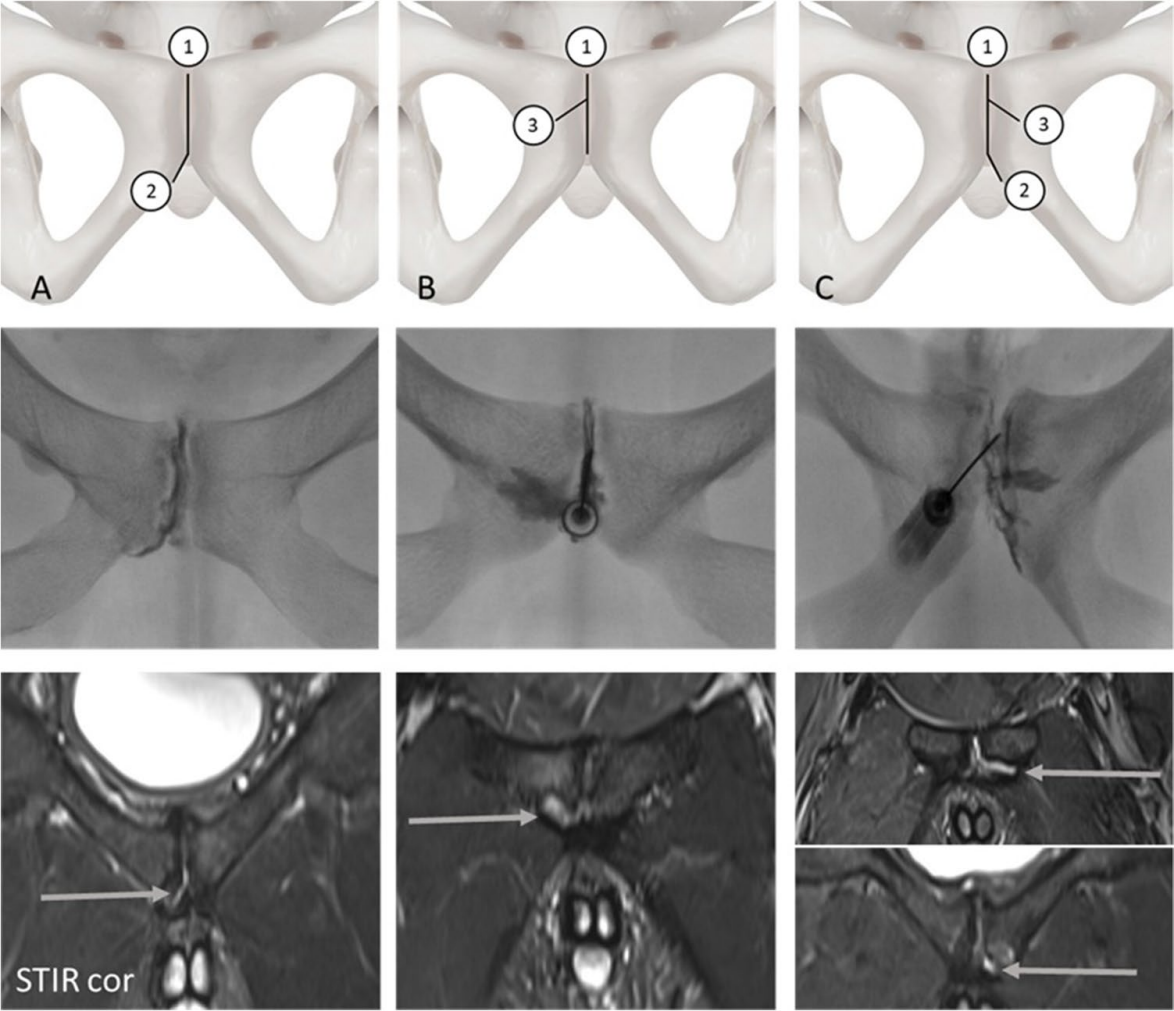
Overview of cleft signs. Upper row: Schematic drawing of cleft signs, second row: fluoroscopic imaging of cleft injuries, third row: MRI examinations represented by a coronal STIR image. Column **A**: isolated secondary cleft, column **B**: isolated superior cleft, column **C**: combined cleft injuries. Arrows highlight cleft signs in MRI examinations. The numbers reflect physiological (primary) cleft (1) and either the secondary cleft (2) or superior cleft (3). Atypical clefts were diagnosed when diagnostic criteria of isolated superior or secondary or combined cleft injuries were not met (see text)

For MRI examinations the diagnostic imaging criteria for superior and secondary cleft signs were adopted as previously described by Byrne et al [2]. Accordingly, the secondary cleft sign is characterized by a linear signal hyperintensity paralleling the inferior margin of the inferior pubic ramus, and the superior cleft sign by a linear signal hyperintensity paralleling the inferior margin of the superior pubic ramus. Both cleft signs had to be in continuity with the physiological primary cleft. Both cleft signs could be present uni- or bilaterally. Additionally, we differentiated between isolated cleft signs and combined (complex) injury patterns, the latter being defined as the presence of superior and secondary cleft signs simultaneously. At last, injury patterns defined as an atypical cleft sign were characterized by a hyperintensity seen in MRI involving the prepubic aponeurotic complex/PLAC (pyramidalis–anterior pubic ligament–adductor longus complex) without meeting the criteria for superior or secondary cleft injuries (Fig. 4). For fluoroscopic imaging, all cleft signs were defined by a characteristic distribution of the contrast agent in accordance to the aforementioned characteristic pattern seen in MRI examinations (Fig. 2). MRI scans and fluoroscopic examinations were reviewed in consensus by two radiologists with 5 years and 22 years of experience. In case of discrepancies between the interpretations, a consensus was found.

Diagnosis of osteitis pubis (OP) required the presence of bone marrow edema (BME) of the pubic body in MR imaging. The area of the affected bone was assessed by visual inspection of coronal STIR images and manual bordering of the maximal area of BME on each side. The slice showing the largest visible area of increased signal intensity in coronal STIR images was selected. Values for the maximal area are expressed in mm^2^ (Fig. 1). A cut-off value of 10% difference (chosen arbitrarily) between sides was defined to determine the dominant side of maximal BME (labeled as BME_10_). Below this cut-off value, the extent of BME was considered to be almost equally distributed between sides.

X-rays in single-legged stance (“flamingo view”) of both sides were carried out in order to assess symphyseal stability. Patients were diagnosed with anterior pelvic ring instability when the vertical shift of the pubic body between sides exceeded 2 mm or a widening of the symphyseal gap greater than 7 mm occurred [1, 13] (Fig. 1).

### Symphyseal injection technique

All Injections were performed using fluoroscopic guidance (symphysography) and under sterile conditions. All patients received a subcutaneous injection of a local anesthetic (0.5 ml bupivacaine) and subsequently 1 ml of a nonionic contrast agent (iomeprol, 300 mg of iodine/ml, Imeron® 300, Bracco Imaging) into the fibrocartilaginous disc of the symphyseal cleft using a 22G lumber puncture needle (Spinocan®, B. Braun Melsungen AG). Needle position was confirmed by fluoroscopic imaging and the presence of the contrasted primary cleft after injection of the contrast agent (compare Fig. 2).

### Analysis and statistics

This study was designed as a prospective observational study. The results should be regarded as descriptive statistics; hence, no *p* values are reported. All data were initially compiled on a Microsoft Excel 2016 spreadsheet. All analyses were performed using JMP software (JMP student, version 16.2.0, SAS Institute Inc.). Data were presented as counts and percentages. We described categorical variables (e.g. type and level of sports) as proportions and where appropriate as percent values and absolute numbers. For quantitative data, the results are expressed as mean ± standard deviation.

## Results

### Demographic factors

A total of 66 male patients were recruited and met the inclusion criteria of the study (Table 1). Patients were most commonly injured while playing soccer (*N* = 56; 85%) with other sports accounting for 15% (*n* = 10) of the injuries. The level of sports varied between patients with 13 patients competing on a professional level (12 × soccer, 1 × ice hockey), 8 on a semi-professional (amateur) level (all soccer) and the other patients competing as recreational athletes. On average the patients completed 4.2 (± 1.6) training sessions per week. The mean duration of groin pain was 12 ± 12.5 months with a range of 1 to 72 months. Treatment for groin pain in advance of the study was administered to 64 patients, most commonly comprising the use of NSAIDs and physical therapy. None of the patients had been treated with a surgical procedure on cleft injuries prior to the study.

### Bone marrow edema and osteitis pubis

The presence of OP as indicated by BME in MRI was seen in 50 patients, bilaterally in 41 patients. Isolated BME without any concomitant injuries (diagnosed in MRI or fluoroscopy) was observed in 3 patients (5%). In 68% (*n* = 28) of patients with bilateral edema, we noted a rather asymmetric distribution with a difference of more than 10% in the total area between sides (BME_10_). Of those 28 patients, 71% (*n* = 20) reported lateralized symptoms on the ipsilateral side of the more pronounced area of BME (Table 3).

**Table 3:** Overview of imaging findings and analysis of side distribution

*n* = 66			
	Total (*n*)	Symptomatic side (*n*)	Side of cleft injury (*n*)
No cleft injury			
MRI	14	Right: 8, Left: 4, Bilateral: 2	
Fluoroscopy	24	Right: 13, Left: 8, Bilateral: 3	
Superior Cleft			
MRI	13	Right: 4, Left: 5, Bilateral: 3, Central: 1	Right: 4, Left: 2, Bilateral: 7
Fluoroscopy	10	Right: 3, Left: 4, Bilateral: 2, Central: 1	Right: 3, Left: 2, Bilateral: 5
Secondary Cleft			
MRI	15	Right: 6, Left: 7, Bilateral: 2	Right: 5, Left: 8, Bilateral: 2
Fluoroscopy	21	Right: 7, Left: 9, Bilateral: 5	Right: 8, Left: 11, Bilateral: 2
combined cleft injuries			
MRI	18	Right: 6, Left: 8, Bilateral: 4	Right: 5, Left: 7, Bilateral: 6
Fluoroscopy	11	Right: 5, Left: 5, Bilateral: 1	Right: 3, Left: 4, Bilateral: 4
Atypical injury pattern			
MRI	6	Right: 4, left: 2	Right: 2, Left: 2, Bilateral: 2
Fluoroscopy	0		
BME			
Total	50		
Bilateral	41		
asymmetric	28	20 ipsilateral to BME_10_; 8 contralateral to BME_10_	
Anterior pelvic ring instability	25		
+ cleft	23		unilateral: 14, bilateral: 9
+ BME	18		

*BME* = bone marrow edema. *BME*_*10*_ = asymmetric BME with side difference in total area  > 10%

### Cleft injuries: MRI vs. fluoroscopy

As one of the main focus points of this study, we compared the presence of cleft signs in MRI and fluoroscopy after injection of iomeprol consisting of 300 mg of iodine/ml as a contrast agent.

In 14 patients we did not find a cleft sign in MRI and none of these patients had a cleft sign in fluoroscopy, either. In contrast, of the 24 patients without a cleft sign in fluoroscopy, 10 patients had a cleft sign in MRI, which were distributed as follows: 2-times isolated superior clefts, 2-times isolated secondary cleft and 6 patients had an atypical cleft sign.

In 13 patients we diagnosed an isolated superior cleft sign in MRI that could also be evidenced in 10 patients in fluoroscopic examinations. In 2 of those 13 patients, we could not find a corresponding cleft sign in fluoroscopy. Additionally, one patient showed an isolated superior cleft sign in MRI and an isolated secondary cleft in fluoroscopy. All 10 patients with an isolated superior cleft sign in fluoroscopy showed this particular injury pattern in MRI, too.

Of the 15 patients with an isolated secondary cleft sign in MRI 13 also showed that injury in fluoroscopy and just 2 did not show any cleft sign in fluoroscopy at all. In comparison, with 21 patients showing an isolated secondary cleft sign in fluoroscopy, MRI examinations of these patients revealed 13 isolated secondary cleft signs, 7 combined cleft signs, and one patient with an isolated superior cleft sign in MRI (same patient as mentioned above).

A total of 18 patients were diagnosed with a combined cleft sign in MRI and of these patients, 11 also were diagnosed with a combined cleft in fluoroscopy. However, the remaining 7 of these 18 patients only showed an isolated secondary cleft sign in fluoroscopy. Of the 11 patients with a combined cleft sign in fluoroscopy, all had a combined cleft sign in MRI as well.

**Fig. 3:**
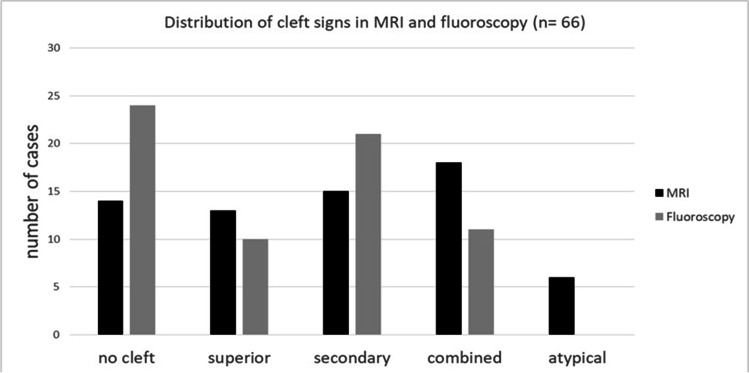
Overview of the distribution of cleft signs in comparison of MRI and fluoroscopic examinations

All 6 cases with an atypical cleft sign could only be depicted by MRI and none were visible in fluoroscopy (Fig. 3).

### Presence of cleft injury, clinical presentation, and anterior pelvic ring instability

In only 4 cases we did neither find a cleft injury in MRI nor a BME. In the majority of cases with a unilateral cleft sign in MRI (*n* = 34; regardless of the type of injury pattern), the side of symptoms matched the side of MRI findings with 76% (*n* = 26) of these patients showing injury patterns in MRI according to their reported side of pain. However, of these 34 patients, 15 patients (44%) also reported their symptoms ipsilateral to the more pronounced side of BME, and in all 15 cases, the unilateral cleft injury was on the same side.

In the cases with bilateral MRI cleft signs (*n* = 17), the reported side of pain was more inhomogeneously distributed with 29% (n = 5) of patients reporting lateralized pain to the left or bilaterally, respectively, and 35% (*n* = 6) to the right. In one case, central pain above the symphysis was reported.

**Fig. 4:**
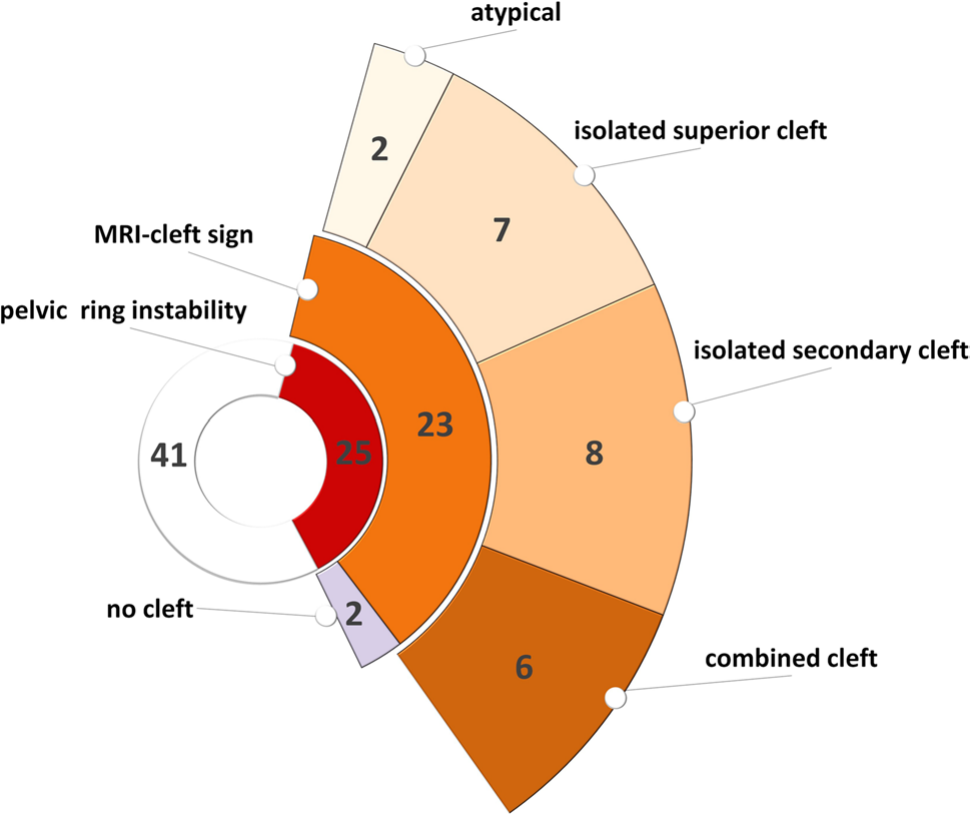
Pelvic ring instability and distribution of cleft signs in MRI

Of all 66 patients 25 (38%) were diagnosed with anterior pelvic ring instability (Fig. 4). Of these patients 23 (92%) were diagnosed with a cleft injury in MRI with a one-sided injury pattern in 14 cases (2 isolated superior cleft, 6 secondary cleft, 4 combined cleft injuries, 2 atypical clefts) and bilateral cleft injuries in 9 cases (5 superior cleft, 2 secondary cleft, 2 combined cleft injuries). Additionally, in 18 of the 23 patients (78%), we observed BME, which was bilateral in 16 of the 18 cases.

## Discussion

In our study we could demonstrate that (1) MRI was superior for diagnosis of cleft injuries when compared to symphysography, (2) the side of injury mostly matched radiological findings, and (3) atypical clefts were not detected in symphysography.

Methodologically, we combined a standardized initial clinical examination by an experienced groin surgeon and subsequent dedicated imaging together with an intrasymphyseal injection for diagnostic reasons. All patients had a history of groin pain typical for cleft injuries. Only in a small number of patients, we could not demonstrate any signs of OP or cleft injuries, supporting the importance of prior clinical examinations as a reference for diagnostic imaging. Our prospective study design allowed a specific definition of the patient´s clinical characteristics regarding symphyseal cleft syndrome. Furthermore, the use of standardized clinical and radiological methods permitted a targeted interpretation.

### MRI was superior for the diagnosis of cleft injuries

Using a standardized dedicated MRI protocol one of the major findings in this study was that MRI proved to be superior in the diagnosis of cleft injuries as compared to fluoroscopic guided contrast agent injection. These findings are in accordance with other studies [1, 14] and further emphasize the role of MRI in the diagnostic process of groin pain. However, our results are in contrast with findings of superior imaging of CT-arthrography for the diagnosis of athletic pubalgia [10] and of symphysography in the detection of cleft injuries [3, 11]. In that regard, there are notable methodological differences between this study and the prior mentioned studies. McArthur et al [10] used a retrospective study design and used a rather small sample size (12 cases). Furthermore, CT-arthrography and MRI were separated by up to 2 months, including the possibility of a healing process in that time span. Additionally, further distribution of contrast agents within the tissue due to the injection technique might have influenced the results (compared to an unenhanced MRI protocol). In the study by Hopp et al [11] also a retrospective design was employed and no specifics on MRI or timing of symphysography relative to prior MRI were disclosed. In comparison, in this study all patients underwent prior specific clinical examinations for cleft injuries, therefore limiting the possible aetiologic spectrum of athletic groin injuries in the first place. Furthermore, all imaging was done within hours using a designated MRI protocol, and as compared to Mc Arthur et al [10], we used a wider spectrum of investigated symphyseal cleft injuries.

Nevertheless, it has to be highlighted that in this study we identified more isolated secondary cleft injuries in symphysography as compared to MRI. Individual case analysis revealed that this effect could be explained by a missed diagnosis of combined cleft injuries in symphysography in those patients, thus resulting in a more frequent diagnosis of combined clefts in MRI due to superior imaging. This view is further supported by our findings of more missed cleft injuries in fluoroscopy as compared to MRI. Only 1 patient had conflicting diagnoses in MRI (isolated superior cleft sign) and fluoroscopy (isolated secondary cleft) that could not be shown in the respective other imaging modality.

### The side of injury mostly matched radiological findings

For diagnosis and radiological grading of OP different radiological methods for evaluation of involved bone are described in the literature. Verall et al [15] graded patients (among other variables) by the size of MRI signal change with a threshold of 2 cm. Branci et al [16] determined the grade of BME according to the distance of the involved bone along the long axis of the superior or inferior pubic ramus. Gaudino et al [12] assessed the extension of BME in the cancellous versus cortical bone. Although in the study presented here, we did not quantitatively rate the severity of BME or OP, our easy-to-use method of assessment of the affected bone area allowed us to discriminate between sides. Using this approach our findings are well in line with previous reports [3, 17, 18] showing rather asymmetric changes and that the leading side of symptoms mostly matched the imaging findings. In detail, in the majority of cases we found either asymmetric patterns of BME in patients with bilateral involvement, ipsilateral cleft injuries according to the leading side of reported symptoms, or a combination of BME and cleft injuries. Isolated BME without any concomitant injuries was a rather rare condition. These findings support those of Cunningham et al [19] and Mosler et al [20] showing that microtearing at the prepubic aponeurotic complex and the presence of BME is a rather frequent cause of groin pain in this kind of population. However, adding to these assumptions, in those cases with bilateral MRI cleft signs we found quite an inhomogeneous clinical presentation.

OP itself might reflect a reaction due to repetitive mechanical stress but could also be detected in asymptomatic athletes [16]. However, as suggested by Garvey et al [21] OP and tearing of adductor muscles might be part of the pathogenesis of acquired pelvic instability. By employing single-legged standing radiography of the pelvis (flamingo view) in this study, we assessed pelvic micro-instability. Of those patients with pelvic instability the vast majority of patients were diagnosed with a cleft injury and to a lesser extent with a BME consistent with OP. We therefore conclude that microtearing at the prepubic aponeurotic complex and the presence of BME are prerequisites for the development of anterior pelvic ring instability. This view is in agreement with the notion that structural deficits (along with functional aspects that were not addressed in this study) play an important role in the pathogeneses of symphyseal instability [21].

### Atypical clefts were not detected in symphysography

**Fig. 5:**
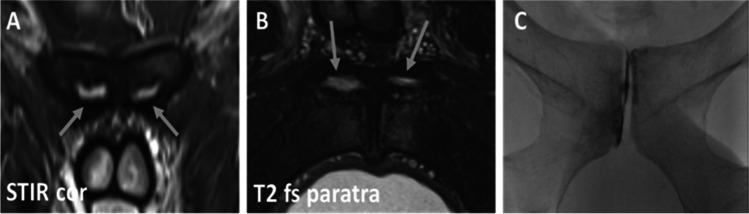
Example of bilateral atypical cleft signs of one patient in MRI (arrows in **A** and **B**) and lack of corresponding pattern in symphysography (**C**). The atypical cleft signs might represent PLAC injuries

Basic definitions of superior and secondary cleft injuries were adapted by Byrne et al [2]. In this study, symphysography failed to identify any of those atypical clefts seen in MRI that were not consistent with the definitions of superior or secondary clefts. According to the anatomical concept of pyramidalis–anterior pubic ligament–adductor longus complex (PLAC) [22] those atypical clefts (Fig. 5) might reflect PLAC injuries [23]. Consequently, considering the possible spectrum of pathologies in athletic groin pain, MR imaging allowed these diagnoses, while symphysography did not.

### Limitations of the study

Some limitations of the study should be taken into account. The study population only involved male patients and there is a lack of an asymptomatic control group. Due to the study design, all patients were pre-screened constituting a selection bias. We have not performed an additional CT arthrography after symphysography due to radiation protection reasons.

## Conclusions

This observational study indicates that dedicated imaging specific to different pathological substrates of athletic groin pain might be beneficial in addition to specialized clinical examination in patients with suspected symphyseal cleft injuries. MRI proved to be superior to symphysography for diagnostic purposes but, as widely accepted, additional therapeutic injections might be beneficial for patients. Additionally, microtearing at the prepubic aponeurotic complex and the presence of bone marrow edema, indicative of osteitis pubis, are considered a prerequisite for the development of anterior pelvic ring instability, and additional Flamingo views should be performed in these patients.

## Abbreviations

BME: Bone marrow edema
cor: Coronal
fs: Fat saturation
NSAIDs: Non-steroidal anti-inflammatory drugs
paratra: Paratransversal
PLAC: Pyramidalis–anterior pubic ligament–adductor longus complex
tirm: Turbo inversion recovery magnitude
tse: Turbo spin echo

## Declarations

### Guarantor

The scientific guarantor of this publication is Dr. Norman Holl.

### Statistics and biometry

No complex statistical methods were necessary for this paper.

### Study subjects or cohorts overlap

Some study subjects or cohorts have been previously reported in a poster presentation at the ESSR congress on post-treatment imaging 2022 in Rostock (Germany).

### Methodology

• prospective

• observational

• performed at one institution
